# Rotavirus Infects Human Biliary Epithelial Cells and Stimulates Secretion of Cytokines IL-6 and IL-8 via MAPK Pathway

**DOI:** 10.1155/2015/697238

**Published:** 2015-07-13

**Authors:** Maria Grazia Clemente, John T. Patton, Robert A. Anders, Robert H. Yolken, Kathleen B. Schwarz

**Affiliations:** ^1^Pediatric Liver Center, Johns Hopkins University School of Medicine, Baltimore, MD 21287, USA; ^2^Pediatric Clinic, Department of Surgery, Microsurgery and Medical Sciences, University of Sassari, 07100 Sassari, Italy; ^3^Virginia-Maryland Regional College of Veterinary Medicine, University of Maryland, College Park, MD 20742, USA; ^4^Department of Pathology, Johns Hopkins University School of Medicine, Baltimore, MD 21287, USA; ^5^The Stanley Division of Developmental Neurovirology, Johns Hopkins School of Medicine, Baltimore, MD 21287, USA

## Abstract

Biliary atresia (BA) is an infantile inflammatory cholangiopathy of unknown etiology although epidemiologic studies and animal models utilizing rotavirus (RV) have suggested a role for viral infection. Proinflammatory and profibrotic cytokines have been detected in infants with BA. The purpose of our study was to investigate the susceptibility of human cholangiocytes (H69 cells) to infection with RRV and to determine if this infection resulted in cytokine secretion. Infection of H69 cells by RRV was noncytolytic and resulted in a time-dependent increase in the release of both infectious virions and cytokines IL-6 and IL-8 into the supernate. The greatest difference in cytokine supernatant levels between infected and mock-infected cells was noted at 24 hours postinfection (h p.i.) for IL-8, 556 ± 111 versus 77 ± 68 pg/mL (*p* < 0.0001), and at 48 h p.i. for IL-6, 459 ± 64 versus 67 ± 2 pg/mL (*p* < 0.0001). Production of both cytokines following RRV infection was significantly reduced by pretreating the H69 cells with inhibitors of mitogen-activated protein kinase (MAPK). *Conclusion*. RRV can infect human cholangiocytes resulting in the production of proinflammatory and profibrotic cytokines via the MAPK pathway. RRV-infected H69 cells could be a useful model system for investigating the viral hypothesis of BA.

## 1. Introduction

Biliary atresia (BA) is a serious infantile liver disease of unknown cause, occurring in ~1 : 5,000–1 : 20,000 live births [1]. We recently studied a group of 289 BA infants enrolled in the Childhood Research and Education Network; 10% had multiple congenital anomalies consistent with laterality defects and 6% had multiple congenital anomalies not of the laterality type [1]. It is assumed that these two forms of BA begin prenatally and are probably genetic in origin [2]. Approximately 65–90% of BA cases are of the perinatal “acquired” type, the so-called “isolated” BA, not associated with major congenital anomalies [1, 3]. Almost certainly the three forms of BA have different etiologies [1]. Given the characteristic time-space clustering and the expression of proinflammatory and profibrotic cytokines both in the liver and in the circulation, including interleukin-6 (IL-6) and interleukin-8 (IL-8), isolated BA is believed by some to be secondary to hepatobiliary viral infection [3, 4]. IL-6 and IL-8 are of particular interest since IL-6 activates macrophages [5] and IL-8 attracts neutrophils [6] and both cell types are present abundantly around the cholangiocytes in the livers of isolated human BA [7, 8]. Huang et al. [9] showed that IL-8 mRNA was increased in livers of BA patients compared to patients with choledochal cyst, both at the time of Kasai hepatic portoenterostomy and later, at the time of liver transplantation. Hepatic IL-6 mRNA was also increased in children in the late stage of BA [9].

Several viruses, including rotavirus (RV), have been implicated as causative agents of BA, but reports have been inconclusive [10]. Extensive investigations by several groups using RRV to induce BA in young mice have focused renewed attention on RV as an etiologic agent of BA [11]. Riepenhoff-Talty et al. [12] first reported RV in hepatobiliary remnants of infants with BA, where they found that 50% of their cohort showed evidence of Group C RV infection [12]. These studies led to experiments which demonstrated that multiple animal RVs could infect Hep G2 liver carcinoma cells [13].

RV is the most common cause of infantile gastroenteritis worldwide, infecting virtually all children by 5 years of age [14]. Although the infection was initially thought to be restricted to the gastrointestinal tract, as RV requires trypsin-like proteases for activation, a number of authors have reported extraintestinal localization of RV in animal models, including RV antigenemia and recovery in multiple organs [15–17]. Furthermore Gilger et al. [18] reported that RV infection of the liver of newborn human infants with immunodeficiency is plausible, perhaps via dendritic cell infection as what has been shown in animal experiments [16]. BA is a rare disease and infants usually present with cholestasis, not with gastroenteritis. RV infection of newborns also is rare, ranging from 5 to 15%, and it is asymptomatic in more than 90% of cases [19]. Thus the rarity of asymptomatic RV infection of the neonate combined with the fact that RV might infect the liver at least provides a logical basis for implicating RV in some cases of human BA.

Therefore, subsequent efforts were made to investigate snap frozen hepatobiliary remnants from children with BA for the presence of Groups A, B, and C RVs [20]. Failure to identify RV in any of these samples led to the conclusion that RV was not commonly involved in the etiology of acquired BA. However, it was later observed that, in mice infected with RRV on the first day of life, the virus cannot be detected in their liver at 2 weeks of age, when the hepatobiliary disease is evident [11], leading us to a reassessment of previous conclusions.

We recently studied the seroprevalence of Group A and Group C RV in infants with BA and cholestatic controls studied during the RV season in the United States (December–May) in the pre-RV vaccine era. The overall prevalence of asymptomatic Group A RV infection found in our study was higher than the 5% previously published rates in this age group. It is of interest that, depending on the sensitivity of the assay used, 10–40% of infants with BA and 18–37% of cholestatic infants without BA did exhibit positive IgM for RV-A [21].

Jafri et al. [22] demonstrated that mouse cholangiocytes were susceptible to RV infection* in vitro* and that inhibition of the mitogen-activated protein kinase (MAPK) family signaling pathway reduced viral replication. Moreover, mouse cholangiocytes respond to RV infection by expressing chemokines* in vitro*, such as MCP-1, RANTES, KC, and MIP-2, some of which have been implicated in the pathogenesis of experimental BA [23]. More recently, the human cholangiocyte H69 cell line was also shown to be susceptible to RV infection in a way that paralleled the murine model of BA [24], providing a human* in vitro* model to further study the pathogenic mechanisms involved in human BA.

Here we show that the human cholangiocyte H69 cell line is susceptible to RV infection* in vitro* and that exposure of the cells to RRV induces the secretion of IL-6 and IL-8, which have been associated with BA in humans. Inhibition of the MAPK family cell signaling pathway significantly reduced the secretion of these cytokines. We confirm that RV infection of human cholangiocytes can be a useful* in vitro* model for investigating the viral hypothesis of acquired BA in humans. Moreover, we provide clear evidence that human cholangiocytes* in vitro* can become immunoregulatory cells in response to virus infection.

## 2. Materials and Methods

### 2.1. Cells and Virus

Rhesus kidney epithelial MA104 cells (ATCC CRL-2378.1) were used to propagate RRV and were grown in Medium 199 containing 5% (vol/vol) fetal bovine serum (FBS), 1% penicillin/streptomycin, and 1% Fungizone (Invitrogen, Carlsbad, CA). Human bile duct epithelial cells (H69 cell line, a biliary epithelial cell line produced from normal human liver) were kindly provided by Drs. N. La Russo and D. Jefferson and were grown as previously described [25].

Prior to infection, RRV was activated by incubation in Leibovitz medium (L-15, Invitrogen) containing 5 *μ*g/mL of porcine trypsin (Sigma, St. Louis, MO) for 30 minutes (min) at 37°C [19]. Cell monolayers were infected at a multiplicity of infection (MOI) of 1 to 5 plaque-forming units (PFUs) per cell. Cells were incubated with viral inoculum for 60 min (MA104 cells) or 90 min (H69 cells) at 37°C. Viral inoculum was replaced with FBS-free Eagle Minimal Essential Medium (MEM) (Invitrogen) containing 0.05 *μ*g of trypsin/mL. RRV titers were determined by plaque assay on MA104 cells [26].

### 2.2. Indirect Immunofluorescence (IF) Assay

Cells were seeded onto coverslips at a density of 5 × 10^4^ cells/well in 6-well plates, infected with RRV or mock-infected, fixed with 4% paraformaldehyde, and permeabilized with 0.5% Triton-X100 in phosphate-buffered saline (PBS). The cells were subsequently incubated with guinea pig anti-RV VP6 polyclonal antisera (1 : 2000 dilution) and, in some cases, mouse anti-cytokeratins 7 and 19 monoclonal antibodies (1 : 1000 dilution). The cells were then incubated with rabbit anti-guinea pig Alexa Fluor 594 (1 : 1000; red signal) alone or with rabbit anti-mouse Alexa Fluor 488 (1 : 1000; green signal). Nuclei were stained with DAPI (4′,6-diamidino-2-phenylindole). Fluorescence was detected with an Olympus BX460 fluorescence microscope (Olympus, Center Valley, PA).

### 2.3. RNA Isolation and cDNA Synthesis

Total RNA was isolated from 1 × 10^8^ H69 cells using a NucleoSpin RNA L kit (Clontech, Mountain View, CA) according to the protocol of the manufacturer. Briefly, H69 cells were detached from culture flasks by incubation with 0.05% trypsin-EDTA, pelleted by low-speed centrifugation, and resuspended in lysis buffer. After homogenization, insoluble debris was removed by centrifugation through filter L columns. After addition of 70% ethanol, RNA was recovered from samples by binding to RNA L columns.

First-strand cDNA was synthesized from RNA using a Superscript III Reverse Transcriptase kit (Invitrogen). Polymerase chain reaction (PCR) amplification was performed using Platinum* Taq* DNA polymerase (Invitrogen) in reaction mixtures containing primers (Table 1) specific for human IL-6, IL-8, MCP-1, TGF*β*1, and GAPDH genes. After 2 min of an initial denaturation at 94°C, cDNAs were amplified under the following conditions: 30 seconds (sec) at 94°C, 30 sec at 56°C, and 1 min at 72°C for a total of 35 cycles (Programmable Thermal Controller PTC-100, MJ Research Inc., Watertown, MA). PCR products were resolved by electrophoresis on 1.5% agarose gels and detected by staining with ethidium bromide and exposing to ultraviolet light.

### 2.4. Quantitative Real Time PCR (qRT-PCR)

qRT-PCR was performed using JumpStart* Taq Ready *Mix (Sigma) and the ABI 7900 HT Fast Real Time PCR system (Applied Biosystem). qRT-PCR cycles included 2 min of initial denaturation at 94°C, followed by 40 cycles of denaturation at 94°C for 15 sec and annealing and extension at 60°C for 1 min. qRT-PCR results were analyzed by the Stratagene Mx4000 Quantitative PCR system. Values were normalized to those obtained for the housekeeping gene, GAPDH (glyceraldehyde-3-phosphate dehydrogenase). For each specific gene, the mRNA relative expression in treated cells was reported as fold difference from or percent (%) of untreated cells.

### 2.5. Human Cytokine-Expression Profile Analysis

Simultaneous detection of 23 human cytokines was performed in culture supernatants from RRV- or mock-infected H69 cells, using the human cytokine antibody array kit (C Series, AAH-CYT-1 kit; Ray Biotech, Norcross, GA; the array map is shown in Table 2). The antibody array membranes were blocked and incubated with 1 mL of undiluted culture supernatant for 2 h. After washing, a cocktail of biotin-conjugated anti-cytokine antibodies was added for 2 h, followed by 1 h incubation with horseradish peroxidase-labeled streptavidin. After final washes, chemiluminescence images were captured and digitized using a laser-based scanner with charge coupled device camera system (Fujifilm LAS-3000, R&D Systems Inc., Minneapolis, MN). Expression levels of cytokines were measured using Fujifilm Multi Gauge software (R&D Systems).

### 2.6. Analysis of IL-6 and IL-8 Levels by Enzyme-Linked Immunosorbent Assay (ELISA)

IL-6, IL-8, and IL-10 levels in the media of mock- or RRV-infected H69 cells at 24 and 48 h p.i. were quantified with Pierce human IL-6, IL-8, or IL-10 colorimetric ELISA kits (Thermo Fisher Scientific, Rockford, IL). The detection range is between 10 and 1000 pg/mL. Samples above the maximum were diluted 1 : 2 in the reagent diluent provided with the kit and retested.

### 2.7. Mitogen-Activated Protein Kinase (MAPK) Pathway Inhibition

To study the effect of inhibition of MAPK pathways on RRV-induced IL-6 and IL-8 secretion, H69 cells were treated with the following MAPK inhibitors: an ERK1/2 inhibitor (U0126, Cell Signaling Technology, Inc., Danvers, MA), a p-38 inhibitor (SB203580, Invitrogen), and a JNK inhibitor (SP600125, EMD Biosciences, La Jolla, CA). All three inhibitors were prepared by dissolving in dimethyl sulfoxide (DMSO) at a concentration of 10 mM and diluted 1000-fold with culture medium. The final concentration of DMSO was 0.1% in all experiments. Cell monolayers were initially incubated for 2 h in L-15 medium containing 10 *μ*M of an inhibitor. Afterwards, the media were removed and the cells were washed with L-15 medium. The cells were then infected with RRV (MOI = 5) or mock-infected for 90 min. The cells were washed 3 times with PBS, and postinfection medium with or without inhibitors (10 *μ*M) was placed on the cells. Levels of IL-6 and IL-8 in cell culture media recovered from mock- or RRV-infected H69 cells at 24 and 48 h p.i. were quantified using Pierce ELISA kits.

### 2.8. Statistics

Experiments were performed in duplicate (immunofluorescence and human cytokine antibody array) and triplicate (ELISA, qRT-PCR, and MAPK inhibition) and were repeated 2 or more times (mock and RRV infection of H69 cells). One-tailed Student's *t*-test was performed to compare the means of two populations. In the bar graphs, data represent the mean ± standard deviation (SD) of multiple repeats. *χ* ^2^ test was performed to compare the values of two populations. A *p* value of less than 0.05 was considered significant. The ^*∗*^ *p* < 0.01 was used to indicate statistical significance of differences between samples.

## 3. Results

### 3.1. Human Biliary Epithelial Cells Are Susceptible to Infection by RRV

**Figure 1:**
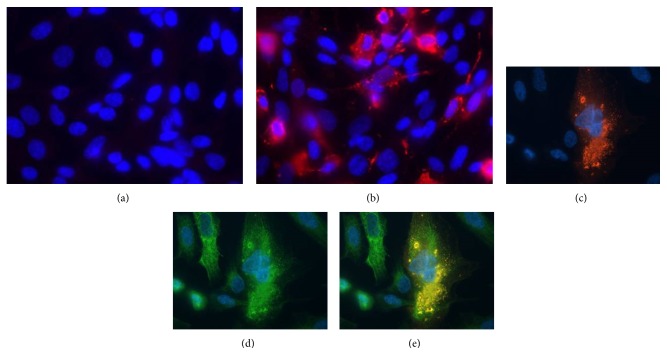
Top panels: immunofluorescence staining of mock- (a) and RRV- (b) infected H69 cells (MOI of 5, 24 h p.i.) using anti-RV VP6 antibody; red cytoplasm fluorescence indicates RRV-infected cells (magnification 40x). Bottom panels: double immunofluorescence staining with anti-RV VP6 (red, c) and CK-7 (green, d) antibodies of RRV-infected H69 cells; (e) is a merged image of (c) and (d). Blue counterstaining of nuclei by DAPI.

Infection of MA104 cells at an MOI of 1 with RRV resulted in extensive cytopathic effects (CPE) and the loss of the cell monolayer by 15 h p.i. In contrast, no cytolysis was obvious in RRV-infected H69 cells at 24 h p.i. at an MOI of either 1 or 5. Moreover, trypan-blue exclusion analysis showed no difference in the viability of mock- and RRV-infected cells at either 24 or 48 h p.i. However, IF assays with RV VP6 antibody revealed the presence of viroplasms in the cytoplasm of infected but not mock-infected H69 cells (Figures 1(a) and 1(b)). Specifically, ~25% of RRV-infected H69 cells infected at an MOI of 5 contained viroplasms. IF assays also showed that the infected H69 cells expressed cytokeratins 7 (Figures 1(c)–1(e)) and 19 (data not shown), confirming their bile duct epithelial histotype (Figures 1(c)–1(e)).

To test whether H69 cells supported productive replication of RRV, supernatants recovered at 2, 24, and 48 h p.i. from RRV-infected and mock-infected H69 cells were analyzed by plaque assay on MA104 cells. The results showed a progressive increase in RRV titers, beginning with 10^2^ PFU/mL at 2 h p.i., reaching 10^6^ PFU/mL at 24 h p.i. and 10^8^ PFU/mL at 48 h p.i., Thus, the H69 cells represent a permissive cell line for RRV growth. This conclusion was further supported by transfer of “postinfection” medium from RRV-infected H69 cells onto MA104 cell monolayers, which resulted in the complete cytolysis of the monolayers upon overnight incubation. As expected, transfer of “postinfection” medium from mock-infected H69 cells to MA104 cells did not result in cytolysis.

### 3.2. RRV-Infected Human Biliary Epithelial Cells and IL-6 and IL-8 Cytokines

**Figure 2:**
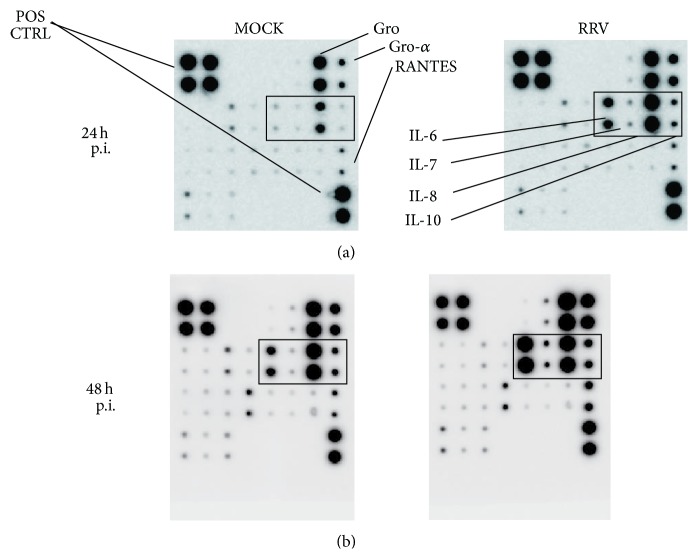
Results of the cytokine array antibody membrane assay: cytokines present in media of mock- and RRV-infected H69 cells at 24 (a) and 48 h p.i. (b). The rectangle in all four membranes indicates IL-6, IL-7, IL-8, and IL-10, the locations of which are indicated in the upper right membrane.

The presence of cytokines in the media of mock-infected and RRV-infected H69 cells at 24 and 48 h p.i. (MOI = 1) was screened using a cytokine antibody array assay (Figure 2). The analysis showed that detectable levels of GRO, GRO-*α*, RANTES, and IL-8 were present in the media of mock-infected H69 cells at 24 h p.i. RRV infection resulted in higher levels of IL-6 and IL-8 accumulation in the media and slightly higher levels of IL-7, IL-10, GRO, and GRO-*α* at 24 h p.i. as compared to mock infection (Figure 2). Likewise, the levels of these cytokines, as well as RANTES, were higher at 48 h p.i. than at 24 h p.i. in RRV-infected-cell media. These results indicate that RRV infection stimulates the expression of some cytokines by H69 cells, notably IL-6, IL-8, and IL-10, which appears to increase overtime.

**Figure 3:**
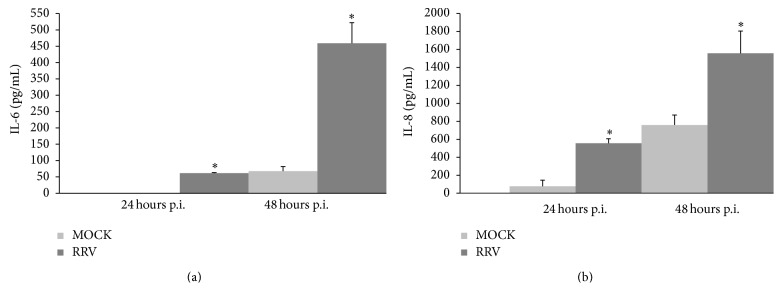
IL-6 (a) and IL-8 (b) detection by ELISA in media of mock- and RRV-infected cells at 24 and 48 h p.i. Bar graphs indicate the mean ± standard deviation (SD) of multiple repeats. Asterisks indicate significant difference between mock and RRV at each time point. ^*∗*^ *p* value <0.01 from mock-infected cells.

**Figure 4:**
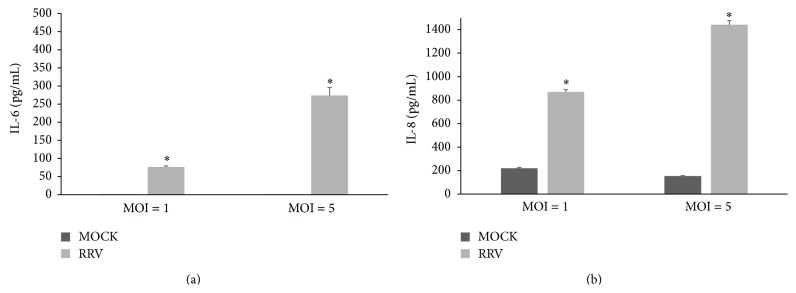
IL-6 (a) and IL-8 (b) detection (pg/mL) by ELISA in media of mock- and RRV-infected H69 cells at 24 h p.i. (MOI = 1 and MOI = 5). Bar graphs indicate the mean ± standard deviation (SD) of multiple repeats. Asterisks indicate significant difference between mock and RRV at each MOI. ^*∗*^ *p* value <0.01 from mock-infected cells.

To validate and quantify the results obtained with the cytokine antibody array assay, the concentrations of IL-6, IL-8, and IL-10 in the media of mock-infected and RRV-infected H69 cells (MOI = 1) were determined by ELISA. As above, the results showed that little or no IL-6 was present in uninfected-cell media at 24 h p.i., with only low levels detectable in the media of such cells at 48 h p.i. (67.4 ± 13.9 pg/mL) (Figure 3(a)). In contrast, infected-cell media contained readily measurable IL-6 levels at 24 h p.i. (61.2 ± 1.9 pg/mL) (*p* < 0.0001 for infected versus mock-infected) and even greater levels at 48 h p.i. (458.8 ± 63.5 pg/mL) (*p* < 0.00020 for infected versus mock-infected). Not unexpectedly, IL-6 levels in the media were dependent on infection conditions, with IL-6 concentrations 3.6-fold higher at an MOI of 5 than at an MOI of 1 in RRV-infected cells at 24 h p.i. (*p* < 0.001; Figure 4(a)) but not in mock-infected cells (Figure 4(a)). At 24 h p.i., IL-6 level was 76 ± 3.1 pg/mL at MOI = 1 versus 273 ± 22.8 pg/mL at MOI = 5 in RRV-infected cells, while IL-6 was undetectable in mock-infected cells at both MOI = 1 and MOI = 5 (Figure 4(a)).

ELISA analysis revealed that, at 24 h p.i., IL-8 levels in the media of RRV-infected H69 cells (556.5 ± 111 pg/mL) were ~7-fold higher than in the media of mock-infected cells (77.0 ± 68 pg/mL) (*p* < 0.0042 for RRV-infected versus mock-infected) (Figure 3(b)). At 48 h p.i., levels of IL-8 in infected-cell media (1558.6 ± 246 pg/mL) were approximately twice as high as that of uninfected-cell media (760.0 ± 49.9 pg/mL) (*p* = 0.011 for RRV-infected versus mock-infected). IL-8 media levels were also influenced by MOI, with levels 1.6-fold higher in the media of H69 cells infected with RRV at an MOI of 5 than 1 (*p* < 0.001; Figure 4(b)). At 24 h p.i., IL-8 level was 870 ± 19.3 pg/mL at MOI = 1 versus 1442 ± 36 pg/mL at MOI = 5 in RRV-infected cells, while IL-8 was 220 ± 7.3 pg/mL at MOI = 1 versus 153 ± 4.6 pg/mL at MOI = 5 in mock-infected cells (Figure 4(b)). IL-10 was not detectable by ELISA in the media of mock- or RRV-infected cells at either 24 or 48 h p.i. (data not shown).

**Figure 5:**
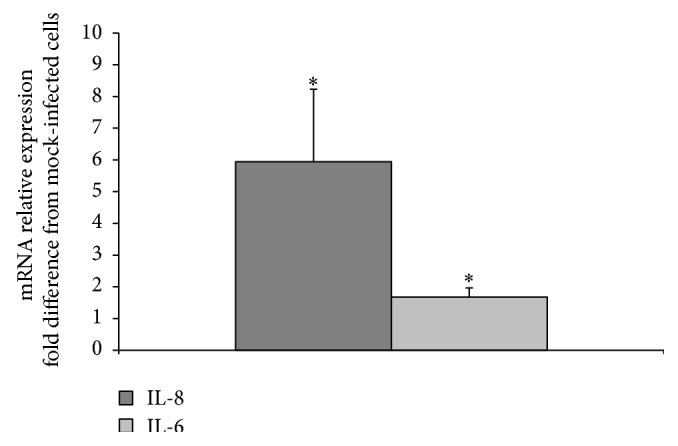
qRT-PCR results: relative expression of IL-6 and IL-8 mRNAs at 24 h p.i. in RRV-infected H69 cells expressed as fold difference from mock. ^*∗*^ *p* value <0.01.

To further evaluate the effect of RRV infection on the expression of cytokines in H69 cells, levels of IL-6 and IL-8 mRNAs in mock and infected cells recovered at 24 h p.i. were determined by qRT-PCR. As shown in Figure 5, IL-6 and IL-8 mRNA levels were approximately 6-fold and 1.5-fold higher, respectively, in infected H69 cells than in uninfected cells. These results are consistent with those presented above (Figures 2–4) which indicates that RRV infection stimulates the expression of IL-6 and IL-8 cytokines by H69 cells. In contrast, qRT-PCR analysis revealed that RRV infection had no impact on the mRNA levels of two other cytokines, MCP-1 and TGF*β*1, used as negative controls in these experiments (data not shown).

### 3.3. Effect of MAPK Inhibitors on IL-6 and IL-8 in RRV-Infected H69 Cells

**Figure 6:**
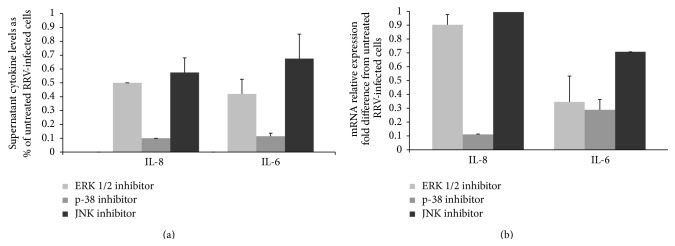
(a) IL-6 and IL-8 levels, measured by ELISA, in the media of RRV-infected H69 cells treated with MAPK inhibitors (p-38, ERK 1/2, and JNK inhibitors), expressed as % of untreated RRV-infected cells. (b) IL-6 and IL-8 mRNA levels, measured by qRT-PCR, in RRV-infected H69 cells treated with MAPK inhibitors (p-38, ERK 1/2, and JNK inhibitors), expressed as fold difference from untreated RRV-infected H69 cells. Bar graphs indicate the mean ± standard deviation (SD) of multiple repeats.

The importance of MAPK activation in the release of IL-6 and IL-8 from RRV-infected H69 cells was examined using the MAPK inhibitors to ERK 2/1, p-38, and JNK. No difference in the viability of mock- and RRV-infected cells treated with the MAPK inhibitors (which were dissolved in 0.1% DMSO) was found by trypan-blue exclusion at either 24 or 48 h p.i. Treatment of RRV-infected H69 cells with SB203580 (p-38 inhibitor) had the greatest effect on IL-6 and IL-8 accumulation in media, as assessed by ELISA, reducing their levels by ~90% (Figure 6(a)). In contrast, U0126 and SP600125 (ERK 1/2 and JNK inhibitors, resp.) treatment reduced IL-6 and IL-8 levels by roughly one-half.

The effect of the MAPK inhibitors on IL-6 and IL-8 mRNA expression was analyzed by qRT-PCR. As shown in Figure 6(b), treatment of RRV-infected cells with SB203580 decreased mRNA expression of both IL-8 (11% of values observed in RRV-infected cells not treated with inhibitor) and IL-6 (28%). When RRV-infected cells were treated with U0126, mRNA expression of IL-8 was 34% of values observed in RRV-infected cells not treated with inhibitor. RNA expression of IL-8 mRNA was not affected when RRV-infected cells were treated with SP600125; mRNA expression of IL-6 was 70% of values observed in RRV-infected cells not treated with SP600125.

Results of the plaque assay experiments showed that the treatment with any of the three MAPK inhibitors did not affect the replication of RRV, as this treatment did not affect the titer of RRV in infected H69 cells.

## 4. Discussion

This study confirms that human biliary epithelial cells are susceptible to productive infection with RRV. RRV infection of human biliary epithelial cells* in vitro* was associated with the increased release of proinflammatory and profibrotic cytokines, such as IL-6 and IL-8, into the media of infected cells. Inhibition of MAPK cell signaling pathway resulted in decreased amounts of both cytokines in the media.

Unlike infection of MA104 cells with RRV, RRV infection of human biliary epithelial cells was not cytolytic but resulted in the release of infectious virions into the cell culture medium, from which infection could be transmitted to MA104 cells with resultant cytolysis. Furthermore, RRV infection of human biliary epithelial cells resulted in a time-dependent increase in the supernatant levels of two proinflammatory and profibrotic cytokines, IL-8 and IL-6. Treatment of the infected cells with a MAPK p-38 inhibitor was associated with a marked reduction in the amount of IL-6 and IL-8 detectable in the supernatant.

The mitogen-activated protein kinase (MAPK) pathway is one of the intracellular signaling pathways that are activated in response to RV infection in different epithelial cell lines, including mouse cholangiocytes [22]. Following RV infection, activation of the MAPK cascade leads to the upregulation of cellular genes. In a human intestinal epithelial cell line (Caco2), RV infection has been reported to be associated with a significant increase in the expression of IL-8 through MAPK p-38 [27]. The promoter of the IL-8 gene has been found to have a binding site for AP-1, which is the last protein to be activated in the MAPK p-38 intracellular cascade [27].

Human cholangiocytes are known to release both IL-6 and IL-8 in response to a variety of injuries* in vivo* [28, 29]. Release of IL-6 occurs when human cholangiocytes are exposed to bacterial lipopolysaccharide [30]. MAPK activity increases in a time- and dose-dependent way in RV-infected mouse cholangiocytes both* in vitro* and* in vivo*, where it is associated with increased production of inflammatory chemokines [22, 23].

Interestingly, as noted above, these two proinflammatory and profibrotic cytokines are thought to be major mediators of tissue damage in acquired BA. Moreover, analysis of BA livers by microarray and qRT-PCR demonstrated prominent expression of proinflammatory genes at early stages of acquired BA, where IL-8 was by far the most upregulated gene (a 17-fold increased expression compared to livers of infants with other cholestatic disorders) [31]. Elevated serum IL-8 has been detected in BA infants with jaundice and/or portal hypertension [32, 33], and the serum concentration of IL-6 has been found to correlate with the severity of BA [34]. IL-6 is released from biliary epithelial cells during liver injury where it directly promotes cell proliferation allowing cell survival and regeneration [35]. Thus IL-6 secretion might represent a means of defense for human biliary epithelial cells to control and to survive the viral infection, explaining why RRV infection did not result in H69 cell lysis* in vitro *[35]. It is interesting to note that the RRV-infected media which resulted in cytolysis in MA104 cells contained both RRV and cytokines. Either IL-6 or possibly other cytokines might have differential effects on cell survival in MA104 and H69 cells or the differential survival of the two cell types in response to RRV infection may be explained by factors other than cytokines.

We should note that our results do not imply a causal role for RV alone in BA especially given our recent observation that the prevalence of RV infection in BA versus other cholestasis disorders (10–40% in BA versus 18–37% in other cholestatic infants) was almost identical [21]. If RV (or any other virus) plays any role in BA, genetically determined differences in immune response to viral infections are likely involved, including differential cytokine responses. As we noted [21] there is substantial precedent for differential host immune responses to infections with other viruses such as hepatitis C virus and HIV.

Our observations that RRV infection of human cholangiocytes involves MAP kinase activation suggests another intriguing avenue by which viral infection might be implicated in the pathogenesis of BA, as prolonged activation of this kinase induced by a viral infection early in life might lead to unbalanced expression of cytokines such as IL-6 and IL-8, which have been associated with progression toward a severe BA phenotype.

In the well-known experimental murine model of RRV-induced BA-like disease, where the hepatobiliary disease progresses in the absence of detectable virus, the potential therapeutic use of MAPK blockers could be explored.

## Figures and Tables

**Table 1:** Primers used for qRT-PCR.

Primer	Sequence
GAPDH, forward	ACAGTCAGCCGCATCTTCTT
GAPDH, reverse	ACGACCAAATCCGTTGACTC
IL-6, forward	TGGAGATGTCTGAGGCTCATT
IL-6, reverse	CGCTTGTGGAGAAGGAGTTC
IL-8, forward	AGCTCTGTGTGAAGGTGCAG
IL-8, reverse	CAGAGCTCTCTTCCATCAGAAA
MCP-1, forward	AGCAAGTGTCCCAAAGAAGC
MCP-1, reverse	TGGAATCCTGAACCCACTTC
TGF*β*1, forward	TTTTGATGTCACCGGAGTTG
TGF*β*1, reverse	GAACCCGTTGATGTCCACTT

**Table 2:** Array map.

	A	B	C	D	E	F	G	H
1	POS	POS	NEG	NEG	G-CFS	GM-CFS	GRO	GRO-*α*
2	POS	POS	NEG	NEG	G-CFS	GM-CFS	GRO	GRO-*α*
3	IL-1 *α*	IL-2	IL-3	lL-5	IL-6	IL-7	IL-8	IL-10
4	IL-1 *α*	IL-2	IL-3	lL-5	IL-6	IL-7	IL-8	IL-10
5	IL-13	IL-15	IFN *γ*	MCP-1	MCP-2	MCP-3	MIG	RANTES
6	IL-13	IL-15	IFN *γ*	MCP-1	MCP-2	MCP-3	MIG	RANTES
7	TGF*β*1	TNF *α*	TNF *β*	BLANK	BLANK	BLANK	BLANK	POS
8	TGF*β*1	TNF *α*	TNF *β*	BLANK	BLANK	BLANK	BLANK	POS

## Abbreviations

BA: Biliary atresia
cDNA: Complementary deoxyribonucleic acid
DAPI: 4′,6-Diamidino-2-phenylindole
ELISA: Enzyme-linked immunosorbent assay
FBS: Fetal bovine serum
GAPDH: Glyceraldehyde-3-phosphate dehydrogenase
h p.i.: Hours postinfection
IF: Indirect immunofluorescence
IL-6: Interleukin-6
IL-8: Interleukin-8
MAPK: Mitogen-activated protein kinase
MEM: Minimal Essential Medium
min: Minutes
MOI: Multiplicity of infection
mRNA: Messenger RNA
PBS: Phosphate-buffered saline
PCR: Polymerase chain reaction
pfu: Plaque-forming unit
qRT-PCR: Quantitative real time polymerase chain reaction
RNA: Ribonucleic acid
RRV: Rhesus rotavirus
RV: Rotavirus
sec: Seconds.
